# Consumer Knowledge, Attitudes and Salt-Related Behavior in the Middle-East: The Case of Lebanon

**DOI:** 10.3390/nu6115079

**Published:** 2014-11-13

**Authors:** Lara Nasreddine, Christelle Akl, Laila Al-Shaar, Mohamad M. Almedawar, Hussain Isma’eel

**Affiliations:** 1 Department of Nutrition and Food Science, Faculty of Agricultural and Food Sciences, American University of Beirut, 11-0236 Riad El Solh, 1107-2020 Beirut, Lebanon; E-Mails: ln10@aub.edu.lb (L.N.); cristell@gmail.com (C.A.); 2 Vascular Medicine Program, American University of Beirut Medical Center, 11-0236 Riad El Solh, 1107-2020 Beirut, Lebanon; E-Mails: la37@aub.edu.lb (L.A.-S.); mma122@mail.aub.edu (M.M.A.); 3 Division of Cardiology, Department of Internal Medicine, American University of Beirut, 11-0236 Riad El Solh, 1107-2020 Beirut, Lebanon; E-Mail: hi09@aub.edu.lb

**Keywords:** dietary salt, consumer, knowledge, attitude, behavior, Middle East

## Abstract

Sodium intake is high in Lebanon, a country of the Middle East region where rates of cardiovascular diseases are amongst the highest in the world. This study examines salt-related knowledge, attitude and self-reported behaviors amongst adult Lebanese consumers and investigates the association of socio-demographic factors, knowledge and attitudes with salt-related behaviors. Using a multicomponent questionnaire, a cross-sectional study was conducted in nine supermarkets in Beirut, based on systematic random sampling (*n* = 442). Factors associated with salt-related behaviors were examined by multivariate regression analysis. Specific knowledge and attitude gaps were documented with only 22.6% of participants identifying processed foods as the main source of salt, 55.6% discerning the relationship between salt and sodium, 32.4% recognizing the daily limit of salt intake and 44.7% reporting being concerned about the amount of salt in their diet. The majority of participants reported behavioral practices that increase salt intake with only 38.3% checking for salt label content, 43.7% reporting that their food purchases are influenced by salt content and 38.6% trying to buy low-salt foods. Knowledge, attitudes and older age were found to significantly predict salt-related behaviors. Findings offer valuable insight on salt-related knowledge, attitude and behaviors in a sample of Lebanese consumers and provide key information that could spur the development of evidence-based salt-reduction interventions specific to the Middle East.

## 1. Introduction

The Middle East and North Africa (MENA) countries represent a region that is now facing a fast rate of development and urbanization, with rates of nutrition-related non-communicable diseases (NCDs) [1] increasing at an alarming rate and exceeding at times those reported from developed countries. It is estimated that, overall, 47% of the region’s burden of disease is due to NCDs, and by 2020 it is expected to rise to 60% [1,2]. In particular, cardiovascular diseases (CVDs) are rapidly growing problems and represent the main underlying causes of morbidity and mortality in the region [1,2,3,4]. There is conclusive evidence that high blood pressure is one of the major risk factors for the development of CVDs [5]. According to the World Health Organization, 49% of coronary heart disease events as well as 62% of all strokes are secondary to high blood pressure [6]. In turn, high dietary salt intake is a well-established risk factor for high blood pressure [7]. Sodium restriction has been suggested to decrease blood pressure in both men and women and in all age groups [8]. Evidence suggests that modest reductions in dietary sodium could prevent serious vascular complications and substantially reduce cardiovascular events and medical costs [9,10]. A recent study conducted in the United States showed that a 3 g reduction in daily salt consumption is associated with a significant decrease in the yearly incidence of coronary heart disease, stroke, myocardial infarction and all-cause mortality, which leads to an estimated decrease in annual healthcare cost by a range of 10 billion to $24 billion [10]. Given this strong body of evidence, the upper limit for sodium intake has been set at 2300 mg/day (close to 6 g of salt per day) by the Institute of Medicine [11] and the World Health Organization (WHO) has called for a reduction in dietary sodium intake to <2000 mg/day (5 g/day of salt in adults), as a cost-effective public health intervention that could potentially reduce the burden of NCDs, including hypertension and CVDs [8]. On a global scale, reducing dietary sodium at the population level has been indicated as the second of the five immediate priority actions for the prevention of NCDs [12].

Strategies to influence dietary sodium consumption include public education designed to alter social norms regarding sodium ingestion, changes to public policy, and improved regulations and food manufacturing practices [13]. Social and cultural factors, along with population age, educational level and average income are primary determinants of dietary behaviors but can be difficult to modify in the short term [14]. Population knowledge and attitudes are thought to influence salt consumption and are considered modifiable mediating factors that are amenable to change [15]. As such, salt reduction efforts often include interventions to raise consumer awareness [16]. In the planning for any intervention to reduce the population’s salt consumption, the WHO recommends assessing the consumer’s attitudes, knowledge, and behavior toward dietary salt as a health risk [17,18,19]. Existing evidence from several developed countries highlights significant gaps in public knowledge related to sodium, its dietary sources, recommended levels of daily intake, and the specific actions required to decrease its consumption [13,20,21,22,23,24]. However, most of the studies investigating consumer’s awareness regarding dietary salt and salt related labels have been conducted in high-income countries and western societies and as such, findings may not be applicable to low and middle-income countries. Among the latter, the Middle East has been largely under-represented, although the region has one of the highest burdens of CVDs and hypertension worldwide. In Lebanon, CVDs account for around 60% of all-cause mortality in persons aged 50 years and older [25] and the prevalence of hypertension has reached 28.8%, a value that exceeds the one reported from the U.S. (18.0%) [26]. Available evidence suggests that the Lebanese population’s sodium intake is high (3.13 g/day), thus highlighting the need to develop and implement national salt reduction interventions [27]. This is a cross-sectional study of adult shoppers in the capital city of Beirut, which aims to (1) establish baseline quantitative data on salt-related knowledge, attitude and behavior (KAB) in a sample of Lebanese consumers aged 18–60 years, and (2) investigate the association of socio-demographic factors, knowledge and attitudes with self-reported salt-related behaviors, namely checking for sodium content on the food label; modifying purchase decisions based on label sodium content; trying to purchase low salt food items; and cutting down on salt. Three of the salt-related behaviors under investigation (checking for sodium content on the food label; modifying purchase decisions based on label sodium content; trying to purchase low salt food items) are pertinent to the consumption of processed, ready-made foods, which have been repetitively shown to be the major contributors to salt intake [5]. In Lebanon, processed foods were found to contribute 67% of the average daily salt intake, with the major contributors being bread & other bread-like products (25%), processed meats (12%) and cheese (10%) [28]. The choice of Beirut as the study site may be justified by the fact that in Lebanon, a Mediterranean country with a territory of 10 452km^2^ and an estimated population of about 4 million, 40% of the population lives in the capital Beirut, which is considered the melting pot of the country [29].

Recognizing that the assessment of knowledge, attitudes and behavior of a population is a crucial component in the development of effective interventions [5], findings generated by this study would provide key information to stakeholders and policymakers on main aspects to be addressed by national salt reduction campaigns in Lebanon and catalyze the development of culture-specific and evidence-based salt-reduction policies that are specific to the Middle East.

## 2. Materials and Methods

### 2.1. Study Design and Subjects

This is a cross-sectional survey of adult supermarket shoppers in the city of Beirut, the capital of Lebanon.

To detect a minimum difference of 12% in the proportion of subjects reporting to adopt favorable behavior between those with positive and negative attitude towards salt, a minimum sample size of 464 is needed, with a power of 80% and within an α error of 0.05. For these calculations, the estimates reported by Grimes *et al*. [22] were used, whereby the behavior of reading salt-related label amongst urban Australian shoppers was found to significantly differ between those with positive and negative attitude towards salt (*i.e.*, whether the subject agrees or not with the statement “My health would improve if I lowered my salt intake”) (75% and 63%, respectively).

The area surveyed (Beirut) was stratified into nine districts based on the administrative classification of the city [29]. One supermarket per district was invited to participate. In case there was more than one supermarket in the district, one was chosen randomly.

We aimed to recruit a total sample of 500 shoppers, stratified over nine supermarkets (55–56 shoppers per shopping supermarket). The survey was conducted by trained nutritionists from January 2012 to January 2013 on different days of the week, including week-ends, and at different times of the day. Adult subjects were invited to participate. Inclusion criteria included: (1) Lebanese nationality (2) reporting to frequently shop for the household and (3) between 18 and 60 years of age. Adults aged above 60 years were not included as older adults may be more exposed to salt-related dietary counseling and medical recommendations given that the likelihood of suffering from diseases, such as hypertension or other CVDs increases with age.

In each supermarket, systematic random sampling was adopted whereby the sampling interval was set at three. The researchers, positioned near the entrances of the shopping centers, approached every third passing shopper and invited him/her to take part in the study [22]. In the case where shoppers declined to participate the researcher would approach the next passing shopper. Oral consent was obtained prior to enrolling in the study. Consenting shoppers filled in a self-administered questionnaire. The study was approved by the Institutional Review Board of the American University of Beirut. The subject's privacy and the confidentiality of the collected data was maintained since data were collected anonymously and without any of the recognized identifiers.

### 2.2. Study Instrument

A multicomponent questionnaire was developed for this study. The first part of the questionnaire inquired about basic sociodemographic information (age category; gender; education level; specialization in a health-related major; and crowding index as an indicator of socio-economic status). The rest of the questionnaire aimed at assessing the consumer’s knowledge, attitude and behaviors, towards salt consumption. The development of the questionnaire was based on a thorough review of the literature and the questions were modeled on those used in past surveys [13,21,22,24,30] but culture-specific modifications were introduced, such as the examples of foods that were included.

The questionnaire was translated to Arabic. The Arabic version of the questionnaire was reviewed by two Arab speaking research nutritionists to ensure that the wording of the questions was culture-specific. The questionnaire was then pilot-tested on a sample of 25 shoppers before its adaptation to field work. Below is a brief description of the various components of the questionnaire.

#### 2.2.1. Knowledge

The knowledge component of the questionnaire inquired about the relationship between high salt/sodium diet and overall health status (improves health, has no effect on health; worsens health; don’t know). Subjects were also asked whether salty foods have an effect on specific health conditions (high blood pressure, stroke, osteoporosis, fluid retention, heart attacks, stomach cancer and kidney disease) by responding “yes”, “no” or “don’t know”. In addition, the participants were asked to identify the statement that best describes the relationship between sodium and salt, the statement that best describes the maximal limit for daily salt intake and the statement that best describes the main sources of salt in the Lebanese diet. Participating subjects were also asked to classify specific foods as high, medium or low in salt/sodium [21].

#### 2.2.2. Attitude

Four questions assessed salt-related attitude. Three questions used a five-point Likert scale ranging from “strongly disagree” to “strongly agree” to assess how important it is for the subject to reduce the amount of salt that he/she adds to foods, the amount of processed foods that he/she eats and the overall intake of sodium [13,21]. For analyses “strongly agree” and “agree” were collapsed into one category to represent agreement with the statement, and “strongly disagree” and “disagree” to represent disagreement [22]. The fourth question inquired whether the participant is concerned with the amount of salt/sodium in his/her diet and to assess (response options: “yes”, “no”) [21].

#### 2.2.3. Behavior

Two questions inquired about the use of food labels in general (frequency of checking food labels; inquiring whether information of food labels affects purchasing decisions) and two questions inquired about the use of salt-related labels in particular (frequency of checking the salt content on food labels; inquiring whether salt-content on the label affects purchasing decision) [21]. Two additional questions determined the frequency of “trying to buy low-salt” products and the frequency of “trying to buy no added salt” foods. Behaviors pertinent to discretionary use of salt were also assessed through questions inquiring about the frequency of adding salt during cooking and at the table [21]. Responses for behavior-related questions included “often”, “sometimes” and “never”. One additional question inquired whether the individual was “cutting down on salt” (response options “yes”, “no”) [30].

In addition to the above sections of the questionnaire, the subject was asked whether he/she thinks that salt-related label information is comprehensible [24], what information on the food package he/she uses to determine how much salt is in the product [30] and how does the subject think his/her salt intake compares to the daily limit of intake [21]. The subject was also asked whether he/she is concerned about artificial flavors, artificial colors, sugar, calories or saturated fat in foods [21].

### 2.3. Data Analysis

Descriptive statistics were performed for demographic variables as well as responses to all questions included in the questionnaire. Continuous and categorical data were expressed as mean (±standard deviation) or counts and percentages respectively. Crowding index, an indicator of socioeconomic status, was calculated as the total number of co-residents per household divided by the total number of rooms, excluding the kitchen and bathrooms [31]. For each individual, a knowledge score was calculated based on the number of correct answers to knowledge questions, with scores ranging from 0 to 27, the higher the score the higher the knowledge. Similarly, an attitude score was created based on the number of favorable attitude statements, with scores ranging between zero and four (the higher the score the more favorable is the attitude towards reducing salt intake). Cronbach alpha coefficients were calculated to determine the internal consistency of the knowledge and attitude questionnaires (knowledge questions were recoded into three different categories to ensure the same measurement scale across all items: correct answer, wrong answer, and don’t know).

Bivariate analyses were done with independent t-tests or chi-square (χ^2^) as applicable. Multivariate logistic regression modeling was conducted and odds ratios and their 95% confidence intervals were reported. All tests with *p*-value of less than 0.05 were considered statistically significant. Data were analyzed using SPSS v.21.0 (SPSS, Chicago, IL, USA).

## 3. Results

### 3.1. Sample Characteristics

A sample of 500 subjects were invited to participate in the study, with 442 subjects (41.6% males; 58.4% females) agreeing to participate (response rate 88.4%). Reasons for refusing to participate included mainly lack of time or disinterest in taking part in the study.

As shown in Table 1, 45.7% of the study subjects were aged between 19 and 30 years. The female to male ratio was of 1.4, with 58.4% women and 41.6% men. The majority of study subjects (91.6%) indicated not having specialized in a health-related major. The study sample was almost equally distributed based on crowding index (47.7% with CrI < 1 person/room and 52.3% with CrI ≥ 1 person/room).

**Table 1:** Socio-demographic characteristics of study participants (*n* = 442) as compared to the distribution of the Lebanese population.

Characteristics	*n*	(%)	Lebanese Population %
**Age (years)**			
19–30	202	(45.70)	31.5 [32]
31–40	105	(23.76)	23.9 [32]
41–50	75	(16.97)	24.1 [32]
51–60	60	(13.57)	20.4 [32]
**Gender**			
Male	184	(41.63)	49.02 [33]
Female	258	(58.37)	50.98 [33]
**Health related major ^a^**			
No	405	(91.6)	-
Yes	37	(8.4)	-
**Educational level**			
Intermediate or lower	23	(5.1)	38.2 [34]
High school or technical degree	115	(26.1)	27.7 [34]
University	304	(68.8)	34.1 [34]
**Crowding index (CrI)**			
<1 person/room	210	(47.7)	37.6 [35]
≥1 person/room	230	(52.3)	62.4 [35]

^a^ Health-related major includes medicine, biochemistry, nutrition, food science, public health, and nursing.

### 3.2. Salt-Related Knowledge, Attitudes and Behaviors in the Study Population

The Cronbach’s α reliability estimate of the knowledge questionnaire was of 0.748. Salt-related knowledge of study participants is shown in Table 2. Overall, the majority of study subjects reported that high dietary salt might worsen health status (77.6%). However, less than one fourth of the study subjects were able to identify processed foods as the main source of salt in the Lebanese diet (22.6%). Similarly, only half of study participants (55.9%) were able to correctly describe the relationship between salt and sodium and less than third of the participants were aware of the 6 g maximum daily limit of salt intake (32.4%) (Table 2). Yet, 63.8% believed their own daily salt intake to be below or equal to dietary recommendations (data not shown).

Gender differentials in salt-related knowledge were noted with a higher proportion of women providing correct answers to several knowledge questions (Table 2). Based on the number of correct answers, a knowledge score was derived for each individual. Accordingly, the mean knowledge score for study participants was of 15.2 ± 4.1, with a significantly higher score amongst women (16.15 ± 3.73) compared to men (13.92 ± 4.15) (data not shown).

**Table 2:** Knowledge related to dietary salt in a sample of Lebanese adult consumers (*n* = 442) by gender.

	Total *n* (%)	Males *n* (%)	Females *n* (%)	*p*-value
**Effect of high salt/sodium diet on health**				
Correct answer (worsen health)	343 (77.6)	121 (65.8)	222 (86.0)	<0.001
**Health problems caused or aggravated by salty foods:**				
High blood pressure	411 (93.0)	167 (90.8)	244 (94.6)	0.122
Stroke	236 (53.4)	91 (49.5)	145 (56.2)	0.161
Osteoporosis	100 (22.6)	35 (19.0)	65 (25.2)	0.126
Fluid retention	235 (53.2)	102 (55.4)	133 (51.6)	0.420
Heart attacks	253 (57.2)	99 (53.8)	154 (59.7)	0.218
Stomach cancer	89 (20.1)	45 (24.5)	44(17.1)	0.056
Kidney disease	248 (56.1)	85 (46.2)	163 (63.2)	<0.001
**Relationship between salt and sodium**				
Correct answer (salt contains sodium)	247 (55.9)	101 (54.9)	146 (56.6)	0.723
**Maximum daily amount of salt recommended for adults**				
Correct answer: (six grams, *i.e*., one teaspoon)	143 (32.4)	56 (30.4)	87 (33.7)	0.467
**Main source of salt in the diet of Lebanese people**				
Correct answer (processed foods)	100 (22.6)	37 (20.1)	63 (24.4)	0.286
**Correctly identified foods as either high, medium or low sources of salt/sodium ^a^**				
Bread (medium)	237 (53.6)	97 (52.7)	140 (54.3)	0.748
Manaeesh-traditional thyme or cheese-filled pies (high)	253 (57.2)	83 (45.1)	170 (65.9)	<0.001
Traditional pies (high)	156 (35.3)	39 (21.2)	117 (45.3)	<0.001
Pizza (high)	227 (51.4)	84 (45.7)	143 (55.4)	0.043
Rice (low)	207 (46.8)	87 (47.3)	120 (46.5)	0.873
Cheese (high)	299 (67.6)	111 (60.3)	188 (72.9)	0.005
Milk (low)	346 (78.3)	140 (76.1)	206 (79.8)	0.345
Pear (low)	357 (80.8)	145(78.8)	212 (82.2)	0.376
Vegetables ragouts (medium)	112 (25.3)	49 (26.6)	63 (24.4)	0.598
Sandwiches e.g., shawarma, fajita, hamburger (high)	331 (74.9)	121 (65.8)	210 (81.4)	<0.001
Soya sauce (high)	329 (74.4)	123 (66.8)	206 (79.8)	0.002
Fresh carrot (low)	336 (76.0)	135 (73.4)	201 (77.9)	0.271
Ketchup (high)	200 (45.2)	68 (37.0)	132 (51.2)	0.003
Bottled salad dressings (high)	228 (51.6)	78 (42.4)	150 (58.1)	0.001
Traditional roasted nuts (high)	373 (84.4)	142 (77.2)	231 (89.5)	<0.001
Sausages and hot dogs (high)	332 (75.1)	121 (65.8)	211 (81.8)	<0.001

**^a^** Correct answers are provided in brackets next to each food item.

The Cronbach’s α reliability estimate of the attitude questionnaire was of 0.724. Salt-related attitude of the study participants is presented in Table 3. Less than half of the study participants (44.7%) stated that they were concerned about the amount of salt in their diet. Based on the number of favorable attitude statements, an attitude score was determined for each participant. Mean attitude score for the study sample was of 2.7 ± 1.2, with a significantly higher attitude score in women (2.96 ± 0.95) compared to men (2.30 ± 1.48) (data not shown).

Participants’ behavioral practices with regard to salt are shown in Table 3. Even though a high proportion of study participants reported that they generally check food labels (67.8%) and that the information on food labels affects their purchasing decisions (66.5%), less than half of participating subjects reported specifically checking for salt content on the food label (38.3%) and that their food purchases are influenced by the salt content (43.7%). In accordance with the aforementioned behavioral practices, only a third of the interviewed shoppers reported trying to buy low salt foods (38.6%). Gender-disparities in behavioral practices were also noted, particularly for checking salt-related labels; modifying purchase decision based on label salt content; and cutting down on salt.

**Table 3:** Salt-related attitude and behavior in a sample of Lebanese adult consumers (*n* = 442) by gender.

	Total *n* (%)	Males *n* (%)	Females *n* (%)	*p*-value
**Attitude**				
You are concerned about the amount of salt/sodium in the diet (Yes)	197 (44.7)	83 (45.4)	114 (44.2)	0.808
Reducing the amount of salt you add to foods is definitely important to you (Agree ^a^)	343 (77.8)	116 (63.4)	227 (88.0)	<0.001
Reducing the amount of processed foods you eat is definitely important to you (Agree ^a^)	356 (80.5)	124 (67.4)	232 (89.9)	<0.001
Reducing your sodium intake is definitely important to you (Agree ^a^)	291 (65.8)	100 (54.3)	191 (74.0)	<0.001
**Behavioral practices **				
*Use of food labels*				
Check labels (Often ^b^)	289 (67.8)	98 (57.6)	191 (74.6)	<0.001
Information on food labels affects purchasing decisions (Often ^b^)	284 (66.5)	94 (55.3)	190 (73.9)	<0.001
Check labels specifically for salt/sodium content (Often ^b^)	164 (38.3)	56 (32.7)	108 (42.0)	0.053
Salt/sodium content indicated on label affects purchasing decisions (Often ^b^)	186 (43.7)	62 (36.5)	124 (48.4)	0.015
Try to buy ”low salt” foods (Often ^b^)	170 (38.6)	55 (30.2)	115 (44.6)	0.002
Try to buy “no added salt” foods (Often ^b^)	89 (20.2)	40 (21.9)	49 (19)	0.46
*Other salt-related behavioral practices*				
Often ^b^ add salt during cooking ^c^	291 (100)	112 (100)	179 (100)	-
Often ^b^ add salt at the table	266 (60.3)	115 (62.8)	151 (58.5)	0.36
Is cutting down on salt intake	235 (53.3)	86 (47.0)	149 (57.8)	0.026

^a^ Attitude was assessed based on a five-point Lickert scale that range from strongly agree to strongly disagree. Answers of “Agree” and “Strongly agree” have been merged. ^b^ Answer options for behavior questions included often, sometimes and never. Answers of often and sometimes have been merged. ^c^ This behavior was assessed amongst those who reported preparing meals for themselves or their families (150 individuals stated that this question was not applicable to them).

### 3.3. Sociodemographic Correlates of Salt-Related Knowledge and Attitude

Female gender was shown to be significantly associated with higher knowledge and higher attitude scores (Table 4). Belonging to a higher age range (>40 years) was also associated with a significant increase in attitude scores, whereas having specialized in a health-related major was significantly associated with a higher knowledge score.

**Table 4:** Sociodemographic correlates of high knowledge ^a^ and attitude ^b^ scores (*n* = 442).

Variables	High knowledge score ^a^		High attitude score ^b^	
	O.R [95% CI]		O.R [95% CI]	
**Age (years)**				
19–30	1.00	(ref)	1.00	(ref)
31–40	1.41	[0.86–2.31]	0.88	[0.53–1.46]
41–50	1.51	[0.88–2.72]	2.29 *	**[1.19–4.41]**
51–60	1.74	[0.94–3.23]	7.12 *	**[2.68–8.94]**
**Gender**				
Male	1.00	(ref)	1.00	(ref)
Female	2.23 *	**[1.48–3.35]**	2.55 *	**[1.64–3.97]**
**Health related major**				
No	1.00	(ref)	1.00	(ref)
Yes	2.62 *	**[1.17–5.87]**	1.99	[0.77–5.14]
**Education level**				
Intermediate or lower	1.00	(ref)	1.00	(ref)
High school or technical degree	0.99	[0.39–2.58]	0.46	[0.15–1.45]
University bachelor’s degree or higher	1.36	[0.55–3.41]	0.69	0.23–2.09]
**Crowding index (CrI)**				
<1 person/room	1.00	(ref)	1.00	(ref)
≥1 person/room	0.84	[0.56–1.25]	1.10	[0.71–1.71]

^a^ Knowledge score is based on the number of correct knowledge answers (scores can range between 0 and 27, the higher the score the higher the knowledge). High knowledge score was defined as ≥75th percentile, which corresponds to 20. ^b^ Attitude score is based on the number of positive attitude statements (scores can range between 0 and 4, the higher the score the more positive is the attitude towards reducing salt intake). High attitude score was defined as ≥75th percentile, which corresponds to three. * Significant at *p* < 0.05.

### 3.4. Association of Sociodemographic Factors, Knowledge and Attitude with Salt-Related Behavior in the Study Sample

Salt-related behavioral practices were significantly associated with specific knowledge questions (Table 5). For instance, those who recognized salt as a dietary factor that can worsen overall health status and those who identified processed food as the main source of salt in the Lebanese diet were more likely to cut down on salt intake. Similarly, those who were aware of the relationship between salt and sodium were more likely to report that their purchase decision is influenced by salt content. As for attitude, shoppers who reported being concerned about the amount of salt in their diet were more likely to adopt all favorable salt-related practices. Similarly, those who have reported a positive attitude towards reducing the amount of salt added to food, the consumption of processed foods and the intake of sodium were more likely to cut down on salt or to modify their food purchase decision based on salt label content (Table 5). Amongst the socio-demographic characteristics, gender and age appeared to be associated with salt-related behavioral practices, with a significantly higher proportion of women and of individuals aged above 51 years adopting favorable salt-related behaviors (Table 6).

**Table 5:** Association of salt-related behaviors with knowledge and attitude (*n* = 442).

	Proportions of Subjects [*n* (%)] * Reporting:							
	Checking label for salt/sodium content		Salt/sodium label content affects purchase decision		Is cutting down on salt		Trying to buy “low salt” foods	
	Often ^a^	Never	Often ^a^	Never	Yes	No	Often ^a^	Never
***Knowledge related to salt***								
**Effect of high salt/sodium diet on health**								
Correct answer	133 (39.9)	200 (60.1)	150 (45.2)	182 (54.8)	**196 (57.3)**	**146 (42.7)**	141 (41.2)	201 (58.8)
Wrong answers	31 (32.6)	64 (67.4)	36 (38.3)	58 (61.7)	**39 (39.4)**	**60 (60.6)**	29 (29.6)	69 (70.4)
**Relationship between salt and sodium**								
Correct answer	104 (43.0)	138 (57.0)	**123 (51.3)**	**117 (48.8)**	133 (53.8)	114 (46.2)	98 (39.7)	149 (60.3)
Wrong answers	60 (32.3)	126 (67.7)	**63 (33.9)**	**123 (66.1)**	102 (52.6)	92 (47.4)	72 (37.3)	121 (62.7)
**Maximum daily amount of salt recommended for adults**								
Correct answer	44 (32.4)	92 (67.6)	52 (38.8)	82 (61.2)	76 (53.5)	66 (46.5)	50 (35.2)	92 (64.8)
Wrong answers	120 (41.1)	172 (58.9)	134 (45.9)	158 (54.1)	159 (53.2)	140 (46.8)	120 (40.3)	178 (59.7)
**Main source of salt in the diet of Lebanese people**								
Correct answer (processed foods)	48 (48.5)	51 (51.5)	48 (48.5)	51 (51.5)	**61 (61.0)**	**39 (39.0)**	48 (48.0)	52 (52.0)
Wrong answers	116 (35.3)	213 (64.7)	138 (42.2)	189 (57.8)	**174 (51.0)**	**167 (49.0)**	122 (35.9)	218 (64.1)
**Health problems caused or aggravated by salty foods**								
High blood pressure								
Yes	151 (37.8)	248 (62.2)	170 (42.8)	227 (57.2)	223 (54.4)	187 (45.6)	159(38.9)	250 (61.1)
No	13 (44.8)	16 (55.2)	16 (55.2)	13 (44.8)	12 (38.7)	19 (61.3)	11 (35.5)	20 (64.5)
Stroke								
Yes	100 (43.7)	129 (56.3)	**114 (50.2)**	**113 (49.8)**	135 (57.2)	101 (42.8)	100(42.4)	136 (57.6)
No	64 (32.2)	135 (67.8)	**72 (36.2)**	**127 (63.8)**	100 (48.8)	105 (51.2)	70 (34.3)	134 (65.7)
Osteoporosis								
Yes	**49 (49.5)**	**50 (50.5)**	**55 (55.6)**	**44 (44.4)**	**64 (64.0)**	**36 (36.0)**	**52(52.0)**	**48 (48.0)**
No	**115 (35.0)**	**214 (65.0)**	**131 (40.1)**	**196 (59.9)**	**171 (50.1)**	**170 (49.9)**	**118(34.7)**	**222 (65.3)**
Fluid retention								
Yes	77 (33.6)	152 (66.4)	86 (37.7)	142 (62.3)	119 (50.6)	116 (49.4)	96(40.9)	139 (59.1)
No	87 (43.7)	112 (56.3)	100 (50.5)	98 (49.5)	116 (56.3)	90 (43.7)	74 (36.1)	131 (63.9)
Heart attacks								
Yes	105 (42.7)	141 (57.3)	120 (49.2)	124 (50.8)	**149 (59.1)**	**103 (40.9)**	105(41.7)	147 (58.3)
No	59 (32.4)	123 (67.6)	66 (36.3)	116 (63.7)	**86 (45.5)**	**103 (54.5)**	65 (34.6)	123 (65.4)
Stomach cancer								
Yes	37 (42.5)	50 (57.5)	41 (47.1)	46 (52.9)	**60 (68.2)**	**28 (31.8)**	37(42.0)	51 (58.0)
No	127 (37.2)	214 (62.8)	145 (42.8)	194 (57.2)	**175 (49.6)**	**178 (50.4)**	133 (37.8)	219 (62.2)
Kidney disease								
Yes	99 (40.6)	145 (59.4)	**117 (48.3)**	**125 (51.7)**	**151 (60.9)**	**97 (39.1)**	109(44.0)	139 (56.0)
No	65 (35.3)	119 (64.7)	**69 (37.5)**	**115 (62.5)**	**84 (43.5)**	**109 (56.5)**	61 (31.8)	131 (68.2)
***Attitude related to salt***								
**You are concerned about the amount of salt/sodium in your diet**								
Yes	**109 (57.1)**	**82 (42.9)**	**117 (61.9)**	**72 (38.1)**	**124 (62.9)**	**73 (37.1)**	**110(55.8)**	**87 (44.2)**
No	**54 (22.9)**	**182 (77.1)**	**68 (28.8)**	**168 (71.2)**	**110 (45.3)**	**133 (54.7)**	**59 (24.4)**	**183 (75.6)**
**Reducing the amount of salt you add to foods is definitely important to you**								
Agree ^b^	142 (42.6)	191 (57.4)	157 (47.3)	175 (52.7)	**219 (63.8)**	**124 (36.2)**	**153(44.7)**	**189 (55.3)**
Neutral	11 (20.8)	42 (79.2)	14 (26.9)	38 (73.1)	**8 (15.1)**	**45 (84.9)**	**9 (17.0)**	**44 (83.0)**
Disagree ^c^	11 (26.8)	30 (73.2)	15 (36.6)	26 (63.4)	**7 (15.9)**	**37 (84.1)**	**8 (18.2)**	**36 (81.8)**
**Reducing the amount of processed foods you eat is definitely important to you**								
Agree ^b^	143 (41.0)	206 (59.0)	161 (46.3)	187 (53.7)	**211 (59.4)**	**144 (40.6)**	154(43.5)	200 (56.5)
Neutral	9 (25.0)	27 (75.0)	11 (31.4)	24 (68.6)	**13 (35.1)**	**24 (64.9)**	7 (18.9)	30 (81.8)
Disagree ^c^	12 (27.9)	31 (72.1)	14 (32.6)	29 (67.4)	**11 (22.4)**	**38 (77.6)**	9 (18.4)	40 (81.6)
**Reducing your sodium intake is definitely important to you**								
Agree ^b^	130 (45.8)	154 (54.2)	**145 (51.1)**	**139 (48.9)**	**196 (67.4)**	**95 (32.6)**	134(46.2)	156 (53.8)
Neutral	16 (19.3)	67 (80.7)	**20 (24.1)**	**63 (75.9)**	**26 (30.2)**	**60 (69.8)**	23 (26.7)	63 (73.3)
Disagree ^c^	18 (29.5)	43 (70.5)	**21 (35.6)**	**38 (64.4)**	**13 (20.3)**	**51 (79.7)**	13 (20.3)	51 (79.7)

^a^ Often and sometimes merged; ^b^ Agree and strongly agree merged; ^c^ Disagree and strongly disagree merged; * Bolded numbers represent significant differences in proportions at p<0.05 (Chi-square test).

**Table 6:** Association of salt-related behaviors with sociodemographic characteristics (*n* = 442).

	Proportions of Subjects [*n* (%)] * Reporting							
	Checking label for salt/sodium content		Salt/sodium label content affects purchase decision		Is cutting down on salt		Trying to buy “low salt” foods	
	Often ^a^	Never	Often ^a^	Never	Yes	No	Often ^a^	Never
***Sociodemographic Characteristics***								
**Gender**								
Males	56 (32.7)	115 (67.3)	**62 (36.5)**	**108 (63.5)**	**86 (47.0)**	**97 (53.0)**	**55(30.2)**	**127 (69.8)**
Females	108 (42.0)	149 (58.0)	**124 (48.4)**	**132 (51.6)**	**149 (57.8)**	**109 (42.2)**	**115(44.6)**	**55 (30.2)**
**Education level**								
Intermediate or lower	8 (34.8)	15 (65.2)	9 (40.9)	13 (59.1)	13 (56.5)	10 (43.5)	11(47.8)	179 (59.1)
High school or technical degree	37 (33.9)	72 (66.1)	43 (39.4)	66 (60.6)	62 (53.9)	53 (46.1)	35(30.7)	79 (69.3)
University bachelor’s degree or higher	119 (40.2)	177 (59.8)	134 (45.4)	161 (54.6)	160 (52.8)	143 (47.2)	124(40.9)	12 (52.2)
**Crowding index (CrI)**								
<1 person/room	78 (37.7)	129 (62.3)	93 (45.1)	113 (54.9)	120 (57.1)	90 (42.9)	86(41.1)	147 (64.2)
≥1 person/room	85 (38.8)	134 (61.2)	92 (42.0)	127 (58.0)	114 (49.8)	115 (50.2)	82(35.8)	123 (58.9)
**Health related major**								
No	151 (38.6)	240 (61.4)	171 (43.8)	219 (56.2)	214 (53.0)	190 (47.0)	154 (38.2)	249 (38.2)
Yes	13 (35.1)	24 (64.9)	15 (41.7)	21 (58.3)	21 (56.8)	16 (43.2)	16(43.2)	21 (56.8)
**Age (years)**								
19–30	**67 (34.4)**	**128 (65.6)**	**83 (42.8)**	**111 (57.2)**	**95 (47.0)**	**107 (53.0)**	**58(28.7)**	**144 (71.3)**
31–40	**36 (35.6)**	**65 (64.4)**	**37 (36.6)**	**64 (63.4)**	**52 (50.0)**	**52 (50.0)**	**40 (38.8)**	**63 (61.2)**
41–50	**30 (40.0)**	**45 (60.0)**	**32 (43.2)**	**42 (56.8)**	**43 (57.3)**	**32 (42.7)**	**35 (46.7)**	**40 (53.3)**
51–60	**1 (54.4)**	**26 (45.6)**	**34 (59.6)**	**23 (40.4)**	**45 (75.0)**	**15 (25.0)**	**37 (61.7)**	**23 (38.3)**

^a^ Often and sometimes merged; * Bolded numbers represent significant differences in proportions at *p* < 0.05 (Chi-square test).

As shown in Table 7, knowledge and attitude scores were significantly higher in subjects reporting to often adopt healthier salt-related behavioral practices as compared to those who reported never adopting such behaviors.

**Table 7:** Mean knowledge and attitude scores (±SD) across the different salt-related behavioral practices in a sample of Lebanese adult consumers (*n* = 442).

	Knowledge Score ^b^	Attitude Score ^c^
	Mean ± SD	Mean ± SD
**Checking label for salt/sodium content ^a^**		
Often	16.04 ± 4.07 *	3.20 ± 1.03 *
Never	14.91 ± 3.87	2.40 ± 1.23
**Salt/sodium label content affects purchase decision ^a^**		
Often	16.00 ± 4.07 *	3.12 ± 1.07
Never	14.84 ± 3.84	2.38 ± 1.24
**Cutting down on salt**		
Yes	16.09 ± 3.76 *	3.20 ± 0.96 *
No	14.22 ± 1.18	2.11 ± 1.26
**Trying to buy “low salt” foods ^a^**		
Often	15.98 ± 3.89 *	3.24 ± 0.94 *
Never	14.74 ± 4.11	2.34 ± 1.28

^a^ Often and sometimes merged; ^b^ Knowledge score is based on the number of correct knowledge answers; ^c^ Attitude score is based on the number of positive attitude statements; * Significant difference between often and never categories for the corresponding behavior (*p* < 0.05).

Table 8 shows the association between salt-related behaviors and sociodemographic characteristics, overall knowledge and attitude scores, based on a multivariate regression model. The results showed that belonging to the age category of 51–60 years increased the odds of trying to buy low salt foods by almost three-fold (OR = 2.61; 95% CI: 1.36–4.99) and that a higher knowledge score was associated with higher odds of cutting down on salt intake (OR = 1.17; 95% CI: 1.01–1.14). Similarly, higher attitude scores were significantly associated with almost a two-fold increase in the likelihood of adopting all of the four salt-related behaviors under investigation.

**Table 8:** Association of Salt-Related Behavioral practices with knowledge, attitude and socio-demographic characteristics (Multivariate regression analysis).

Variables	Often Checking Label for Salt/Sodium Content ^a^		Salt/Sodium Label Content often Affects Purchase Decision ^a^		Is Cutting Down on Salt		Often Trying to Buy “Low Salt” Foods ^a^	
	O.R. [95% CI] *		O.R. [95% CI]		O.R. [95% CI]		O.R. [95% CI]	
**Gender**								
Male	1.00	(ref)	1.00	(ref)	1.00	(ref)	1.00	(ref)
Female	1.07	(0.67–1.69)	1.24	(0.79–1.94)	0.86	(0.55–1.40)	1.23	(0.78–1.97)
**Age (years)**								
19–30	1.00	(ref)	1.00	(ref)	1.00	(ref)	1.00	(ref)
31–40	0.94	(0.55–1.61)	0.64	(0.38–1.09)	1.09	(0.63–1.87)	1.61	(0.93–2.79)
41–50	0.81	(0.45–1.49)	0.62	(0.34–1.12)	0.87	(0.47–1.60)	1.46	(0.80–2.65)
51–60	1.46	(0.76–2.81)	1.24	(0.64–2.39)	1.71	(0.85–3.47)	**2.61**	**(1.36–4.99)**
**Education level**								
Intermediate or lower	1.00	(ref)	1.00	(ref)	1.00	(ref)	1.00	(ref)
High school or technical degree	1.17	(0.42–3. 82)	0.45	(0.45–3.43)	1.24	(0.43–3.54)	0.57	(0.20–1.59)
University bachelor’s degree or higher	1.58	(0.59–4.22)	0.60	(0.60–4.16)	1.06	(0.39–2.88)	0.91	(0.34–2.41)
**Crowding index (CrI)**								
< person/room	1.00	(ref)	1.00	(ref)	1.00	(ref)	1.00	(ref)
≥1 person/room	1.18	(0.77–1.82)	0.99	(0.65–1.51)	0.77	(0.50–1.19)	0.91	(0.59–1.41)
**Knowledge score ^b^**	1.04	(0.98–1.10)	1.04	(0.98–1.10)	**1.17**	**(1.01–1.14)**	1.02	(0.96–1.08)
**Attitude score ^b^**	**1.82**	**(1.47–2.26)**	**1.67**	**(1.37–2.05)**	**2.22**	**(1.79–2.75)**	**1.93**	**(1.54–2.42)**

^a^ Often and sometimes merged; ^b^ Knowledge and attitude scores were considered as continuous variables; * Bolded numbers are significant at *p* < 0.05.

## 4. Discussion

This study has explored salt-related knowledge and attitudes and the impact of these constructs on self-reported behaviors in a sample of adult shoppers in Lebanon, a country of the Middle East region where CVDs and hypertension rates are exceeding those reported from other parts of the world [1]. Our results suggest that consumer awareness about salt in Lebanon is rather low and that a small proportion of consumers regularly adopt behavioral practices that help to reduce their salt intake. The study’s findings cannot be compared to previous studies in Lebanon or the Middle East as such investigations are lacking. However, the study showed that salt-related knowledge and attitudes amongst urban Lebanese consumers are similar to those reported from developed countries [16,21,22,24,36]. In fact, the majority of consumers were aware that high salt intake may be associated with adverse health effects and that high blood pressure can be caused by excessive salt consumption. However, and in agreement with previous studies, many participants were unaware of the other health conditions that are potentially associated with high sodium intake [16,22,24,36]. Similarly, the study showed that most participants were unsure of the maximal limit for salt intake, while at the same time, most respondents believed their own daily salt intake would be equal to or below the maximal limit for salt consumption. These findings are in line with those reported by Land *et al*. [16] and Grimes *et al*. [22] amongst adult shoppers in Australia, those reported for populations in five countries in the Americas (Argentina, Canada, Chile, Costa Rica and Ecuador) [37] and those reported from Greece [38].

The study’s findings have also shown that most participants had limited knowledge of the main foods that contribute to salt in the diet, with only a quarter of the study population identifying processed foods as the main contributors to salt intake. This is in stark contrast with data stemming from a nationally representative food consumption survey in Lebanon (2009), whereby processed foods were found to contribute 67% of the average daily salt intake [28]. These findings suggest that, as reported by previous consumer studies conducted in other parts of the world, Lebanese consumers are unaware that some of the main sources of dietary salt in the diet are ‘hidden’ in everyday food items [21,22,37,38,39]. This may explain why less than half of the study participants stated that they were concerned about the amount of salt in their diet. Interestingly, the proportion of participants reporting to be concerned about dietary salt (44.7%) was lower than the proportion reporting to be concerned about the amount of saturated fat (64.4%), artificial flavors (60.5%), calories (60.1%), artificial colors (57.4%) and sugar (55.5%) in the diet (data not shown). These findings suggest that salt falls low on the list of health priorities amongst Lebanese consumers.

The study’s findings showed that, while close to two-thirds of the study participants reported to regularly check food content labels, only a third reported checking specifically for salt content. More alarmingly, greater than half of the study population reported that the salt content on the label doesn’t affect their decision to purchase the product. These findings are in agreement with those reported amongst a sample of Australian adults [40] and represent a further attestation to the fact that salt is not perceived as much of a health risk as other food constituents and ingredients. Alternatively, the low proportion of participants reporting to check salt-related label content may be an artifact of the consumer’s difficulty in using and interpreting labeled sodium information, whereby the majority of study participants (62.2%; data not shown) stated that salt-related label information is not comprehensible.

The results obtained in this study identified older age and female gender as characteristics associated with higher salt-related knowledge and a more favorable attitude towards salt reduction. Similar to our findings, other studies confirmed that older people tend to demonstrate higher levels of nutrition knowledge [41,42], and specifically higher salt-related knowledge and awareness [40]. Older adults may in fact be more exposed to dietary counseling and nutritional/medical recommendations given that the likelihood of suffering from NCDs increases with age. The observed association with female gender is in line with other studies reporting women to have higher salt-related awareness, to be more health-conscious and to more readily follow dietary recommendations than men [40,43,44].

According to the theory of planned behavior (TPB), intention is an immediate precursor to behavior, and in turn, intention is influenced by knowledge, attitudes, subjective norms and perceived behavioral control [45,46,47]. In this study, we have investigated knowledge and attitude as predictors of salt-related behavior. Accordingly, higher salt-related knowledge was found to be significantly associated with “cutting down on salt”. More specifically, individuals who were aware of the effects of high dietary salt on health were more likely to report cutting down on salt. In addition, individuals who were aware of the relationship between salt and sodium were more likely to modify their food purchase decision based on salt label content. These findings are in agreement with those provided by several previous cross-sectional studies, which have shown that knowledge is associated with favorable salt-related behavioral practices [9,22,48].

In the present study, consumers’ attitude towards salt was found to be associated with all of the four salt-related behaviors under investigation, including using salt-related label, modifying purchase behavior based on salt content, cutting down on salt and trying to buy low salt foods. More specifically, and in agreement with findings reported by Grimes *et al*. [22], individuals who reported being concerned with the amount of salt in their diet were more likely to adopt favorable salt-related behaviors.

It is acknowledged that this study has a number of limitations. Similar to several previous published studies [22,24], the study was restricted to an urban setting and therefore the findings may not be extrapolated to the Lebanese population as a whole. Even though the distribution of the study sample in terms of gender was relatively similar to the Lebanese population distribution [33], the proportion of younger adults (19–30 years) was over-represented in the study sample at the expense of those aged above 41 years [32] (Table 1). Previous studies have suggested a negative relationship between age and response rate in surveys [49] and a linear decline of response rate with age [50]. Similarly, the proportion of subjects with university education in the study sample is higher than the one reported for Lebanon [34]. It is possible that university education is more commonly encountered in the urban setting of the capital Beirut, compared to other areas in the country. It is also possible that those with a higher educational level are more likely to accept to participate in a survey. In a study that investigated the characteristics of responders and non-responders in a health survey, response rate was found to increase with increasing education level [51]. In agreement with the over-representation of those with higher educational levels, the proportion of subjects with high socioeconomic status (SES), based on crowding index, was also over-represented in the present study (Table 1). However, despite overrepresentation of those with university level and those of higher socioeconomic background, still the majority of participants had difficulty in interpreting the current sodium labeling information and pinpointing the main dietary contributors to sodium intake. It may therefore be expected that salt-related awareness is even lower amongst consumers from lower education and SES backgrounds, and that the results of this study may have provided an overestimate of consumer’s salt-related knowledge and attitude in Lebanon. In agreement with several previous studies [22,37,38], this study did not use a validated questionnaire as none was available, but the questionnaire was modeled on those used in past surveys [13,21,22,24,30] and it was pilot-tested. In addition, and also in agreement with other studies [22,37,38], the survey was based on self-reported behavior, which may be different from actual behavior. Assessment of actual consumers’ behavior would require shadowing and direct observation. Alternatively, it was suggested that the assessment of 24-hour urinary excretion of sodium might be an indirect measure of salt-related behavior. However, this approach may be limited by the significant within person variation in salt consumption from day to day and considerable participants’ burden [16]. Finally, subjects were not asked whether they were suffering from hypertension or had previously received dietary/medical consultations for a low salt diet. The sensitive nature of medical data may have directly influenced the response rate in the study. However, the selection criteria of 18–60 years old may have limited the number of adults suffering from chronic diseases in the study sample.

Despite the limitations of the study, the findings presented in this paper provide valuable insight on salt-related knowledge and attitude in a sample of Middle Eastern consumers, providing key information that could guide the development of effective, evidence-based salt-reduction programs and inform adequate policies. The identified knowledge and attitude gaps gain all the more importance as these attributes were found to be significant predictors of salt-related behavior in the study sample. Therefore, the study’s findings as a whole echo the recommendations of the WHO, which call for intervention strategies and educational campaigns aiming at increasing consumer’s knowledge and awareness [17,18,19]. For instance, raising awareness about the maximal limit of daily salt intake and about the relationship between salt and sodium may help the consumer make better informed choices when purchasing processed foods and facilitate the understanding of nutrition information on food labels [38]. Increasing consumer’s awareness and highlighting processed foods as the main source of dietary salt may not only help the consumer make better informed choices but may also exert pressure on the food industry to take actions towards lowering the sodium content of food [38]. Intervention strategies may rely on the use of the media as an awareness vehicle, as it is recognized that attitudes, social norms, and cultural opinions about food, eating and dietary choices are partially shaped by the media including magazines, television, videos, and the Internet [52]. Examples of actions that can be adopted in the local context of Lebanon to increase consumer’s knowledge and foster a favorable attitude towards salt reduction may rely on collaborating with the press and the media to disseminate simple and coherent messages regarding salt. Other examples may include engaging renowned chefs to cook on television with less salt while proposing alternatives to enhance flavor and taste. Similarly, local initiatives aiming at familiarizing the consumer with food label information or even at simplifying salt-related information on food labels such as a clear indication of “high in salt” may also be recommended, as has been successfully implemented in Finland [38,53].

## 5. Conclusions

This study highlights specific gaps in salt-related knowledge, attitude and behavioral practices in a sample of Middle-Eastern adults, thus emphasizing the need for culture-specific education and awareness campaigns on salt, its dietary contributors and its association with health.

While there is strong evidence regarding the impact of policies that target the food industry and the salt environment as a whole, there is also evidence that shows that nutrition education and consumer awareness have the potential to effectively bring behavior change around salt [17]. “Although policies and programs can make healthy options available, people still have the responsibility to make healthy choices. People are empowered when they have the knowledge, ability, resources, and motivation to identify and make healthy choices” [54] (p. 22). As of 2010, at least 32 countries around the world had implemented salt reduction initiatives, the vast majority of which (28) included nutrition education and public awareness campaigns [55]. In this context, the results of this study should provide the backbone for the development of intervention strategies aiming at increasing salt-related consumer awareness in Lebanon and could serve as a stepping-stone in spurring the development of policies tailored to the Arab region.
